# Using IEA International Civic and Citizenship Education Study (ICCS) Data: Northern Lights on ICCS

**DOI:** 10.1007/978-3-030-66788-7_1

**Published:** 2021-02-27

**Authors:** Heidi Biseth, Bryony Hoskins, Lihong Huang

**Affiliations:** 19 Department of Culture, Religion and Social Studies, University of South-Eastern Norway, Drammen, Norway; 20 Department of Social Sciences, University of Roehampton, London, UK; 21 Norwegian Social Research--NOVA, Oslo Metropolitan University, Oslo, Norway; 22 Department of Culture, Religion and Social Studies, University of South-Eastern Norway, Drammen, Norway; 23 Department of Social Sciences, University of Roehampton, London, UK; 24 Norwegian Social Research—NOVA, Oslo Metropolitan University, Oslo, Norway

**Keywords:** Nordic context, Country specific findings of ICCS, Understanding of civic engagement, Democratic dispositions, Inequalities, International Civic and Citizenship Education Study (ICCS)

## Abstract

This chapter introduces the Nordic context of civic and citizenship education in schools including reviews of previous results and research on IEA’s International Civic and Citizenship Education Study (ICCS). By discussing the issues relevant to democratic citizenship education that are of central significance in the four Nordic countries, this chapter argues for new cross-country comparative analyses of ICCS data based on themes typically engaging Nordic scholars, including students’ understandings of citizenship, school principals’ understandings of the priorities of citizenship education, digital citizenship education, environmental citizenship education, and inequalities and citizenship education. Furthermore, this chapter provides a layout of the volume through positioning the five analytical chapters across *contesting the understanding of civic engagement* and *democratic dispositions in Nordic democracies.*

## Introduction

IEA’s (International Association for the Evaluation of Educational Achievement) International Civic and Citizenship Education Study (ICCS) is the only regular dedicated comparative international study of civic and citizenship education. In 2009 and 2016, national representative samples of grade 8 (grade 9 in Malta and Norway) students from educational systems across the world participated in the study, including four of the Nordic countries: Denmark, Finland, Norway, and Sweden. In addition to an international assessment and survey, regional modules have been administered in Europe and Latin America. The various reports from the ICCS study provide a detailed overview of the study’s results (e.g., Losito et al. 2018; Schulz et al. 2018; Huang et al. 2017; Bruun et al. 2017, 2018; Skolverket 2017; Mehtäläinen et al. 2017; Finnish Institute of Education Research 2017). The ICCS 2016 and 2009 studies built on a history of IEA citizenship studies (the Civic Education Study [CIVED] 1999, and the Six Subject Survey conducted in 1971). Having had two cycles that used the same framework within ICCS has enabled researchers to monitor trends in civic knowledge and engagement over seven years for the countries that participated in both ICCS 2009 and ICCS 2016.

The ICCS studies investigate the ways in which young people are prepared to undertake their roles as citizens in a world where contexts of democracy and civic participation continue to change. It reports on students’ knowledge and understanding of concepts and issues related to civics and citizenship, as well as their beliefs, attitudes, and behaviours concerning this domain. The study collects a rich array of contextual data about the organization and content of civic and citizenship education in the curriculum, teacher qualifications and experiences, teaching practices, school environment and climate, and home and community support.

In this book, we present a Nordic comparative study on civic and citizenship education with a focus on the themes of: Nordic students’ understandings of citizenship, Nordic principles’ understandings of the priorities of citizenship education, digital citizenship education, environmental citizenship education, and inequalities in citizenship education. Nordic countries have a long history of democracy, equality, and human rights (see e.g., Ringen 2007, 2011; Economist Intelligence Unit [EIU] 2020), and this model is seen as providing an example, a “northern light,” that many countries may be interested to learn from. There is considerable interest in the Nordic models of education and the young people’s attitudes, values, civic knowledge, and skills that can be seen to be formed from these education experiences. This book will shed light on citizenship learning and identify the extent that there is a common Nordic model on civic education and young people’s citizenship competences and how they are changing over recent years.

## The Nordic Context

The benefit for comparative research is that Nordic countries are similar in many respects that make them apt for comparison. These countries combine relatively small populations, high Gross National Income (GNI) per capita, and long life expectancy (see Table 1.1). What is more, the histories of the countries are closely intertwined with close political collaboration across borders and all countries having been peaceful since the Second World War. We recognize that this is painting a picture with broad strokes, as differences do exist. For example, Finland’s proximity to Russia has had an impact on its policies after the Second World War which make it different from that of the other Nordic countries. While Denmark, Norway, and Sweden have languages mutually comprehensible, this is not the case for the Finnish language. Nevertheless, due to historical reasons, many Finns can speak Swedish and both languages are official languages in Finland (Hult and Pietikainen 2013). Finland, Norway, and Sweden also have a Sami indigenous population, speaking several Sami languages which are in the same language group as the Finnish language (see e.g., Lindgren et al. 2016). Moreover, despite all countries having relatively small populations, Sweden is about double the size of each of the other three countries. All the countries have a high GNI per capita, but Norway has a significantly higher GNI than the other three countries (see Table 1.1).

**Table 1.1:** Selected demographics and economic characteristics of the Nordic countries participating in the ICCS 2009 and 2016 studies

	Population (in thousands)	Human Development Index					Democracy Index
		Value	Rank	Life expectancy	Mean years of schooling	Gross National Income (GNI) per capita in USD $	
Denmark	5,797.45	0.930	11	80.8	12.6	48,836	9.22 (rank 7)
Finland	5,518.05	0.925	12	81.7	12.4	41,779	9.25 (rank 5)
Norway	5,314.34	0.954	1	82.3	12.6	68,059	9.87 (rank 1)
Sweden	10,183.17	0.937	8	82.7	12.4	47,955	9.39 (rank 3)

*Sources* Data on Human Development Index and GNI per capita obtained from the *UNDP Human Development Report 2019* (UNDP 2020). Data on population size sourced from *World Bank Open Data 2018* (World Bank 2019). Data on Democracy Index obtained from the *EIU Democracy Index 2019* (EIU 2020)

What is important and interesting for this book, is that Nordic countries have long-standing traditions as democracies, with social democratic models of society (see e.g., EIU 2020; Ringen 2007, 2011; Wiborg 2004). The four Nordic countries are ranked among the top 10 in the Democracy Index based on the five categories: electoral process and pluralism; civil liberties; the functioning of government; political participation; and political culture (EIU 2020, and see Table 1.1). Denmark and Norway are both in the top five of countries according to the level of satisfaction of their population with democracy in the world and Sweden and Finland are ranked in the top 10 (EIU 2020).

The four Nordic countries score among the top 12 out of 189 states ranked in the Human Development Index (HDI) (UNDP 2020). This index measures (1) access to a decent standard of living through a country’s GNI per capita, (2) access to knowledge through mean number of years of schooling and expected years of schooling, and (3) the potential for a long and healthy life through life expectancy at birth (UNDP 2020).

The education sector is the one institution in society with which the Nordic population is well acquainted since all spend a decade of their early lives in compulsory education, and have the choice to participate in higher and adult education as it is made readily available and free for all (UNESCO 2020, pp. 286–287, 296–297, 312). The four Nordic countries discussed here all have a high relative expenditure on education ranging from 7.2 to 8.5% of GNI (UNESCO 2020, p. 287, see also Schulz et al. 2018, pp. 46–47).

In Table 1.1 we present a selection of demographic and economic characteristics of the four Nordic countries participating in the ICCS 2009 and 2016 studies.

The fact that the countries are quite similar regarding many of these international standards/rankings provides a solid basis for comparison as significant differences on ICCS scores are more easily attributed to specific policy differences. In addition, general high levels of wealth and levels of education of parents, which are suggested by these country rankings, are argued to be the foundations for young people to have more cosmopolitan and social justice related attitudes and values (Inglehart 2007) so we could already expect results to be above the international mean for young Nordics on these scores.

## Nordic Results in ICCS

IEA’s ICCS measures three main components, namely (1) civic knowledge, (2) civic engagement, and (3) civic attitudes among 14-year-olds. The four Nordic countries score among the top five on civic knowledge (Schulz et al. 2018, p. 58). However, it is worth mentioning that civic knowledge varied more within than across countries (Schulz et al. 2018, p. xvii). In general, girls have higher civic knowledge scores than boys and this is the case for all Nordic countries. As shown in Table 1.2, the Danish boys outperform boys from all other countries in the study on civic knowledge in both ICCS 2009 and ICCS 2016 studies. However, Finnish girls outperform on civic knowledge achievement from all girls in ICCS 2009 while Swedish and Danish girls outperform from girls in Finland and Norway in ICCS 2016 (Bruun et al. 2017, 2018; Huang et al. 2017).

**Table 1.2:** Civic knowledge achievement of boys and girls and changes between ICCS 2009 and ICCS 2016

	Boys		Girls		Total average		Points change from 2009 to 2016	
	2009	2016	2009	2016	2009	2016	Boys	Girls
Denmark	573 (4.5)	575 (3.7)	581 (3.4)	597 (2.9)	576 (3.6)	586 (3.0)	2 (5.9)	16 (4.3)*
Finland	562 (3.5)	561 (3.4)	590 (2.9)	594 (2.3)	576 (2.4)	577 (2.3)	−1 (4.9)	4 (3.6)
Norway	527 (4.6)	547 (2.6)	552 (4.5)	581 (2.4)	538 (4.0)	564 (2.2)	20 (5.3)*	29 (5.6)*
Sweden	527 (4.2)	562 (3.9)	549 (3.4)	598 (3.1)	537 (3.1)	579 (2.8)	35 (5.4)*	49 (4.5)*
International average	489 (0.7)	505 (0.8)	511 (0.7)	530 (0.8)	500 (0.2)	517 (0.2)	16 (1.1)*	19 (1.1)*

*Notes* All calculations are performed using the IEA IDB (International Database) Analyzer applying student weight. Numbers in parenthesis are standard errors.

*indicate a change is significant at 0.05 level.

**Fig. 1.1:**
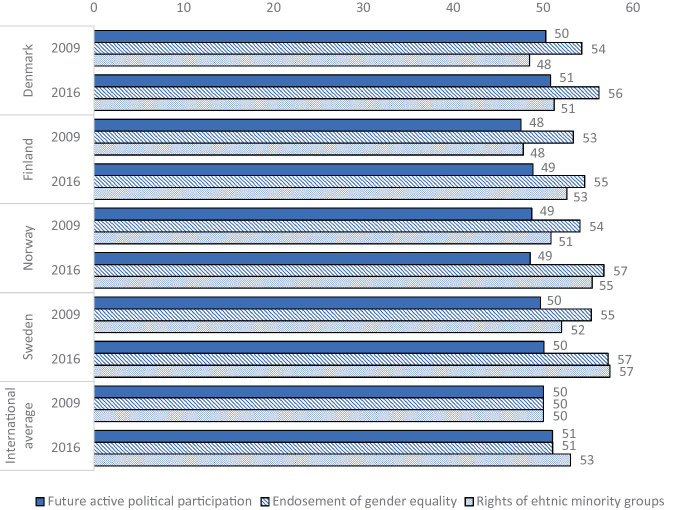
Nordic student civic attitudes and future political participation scales in comparison with international averages of ICCS 2009 and ICCS 2016 (*Notes* Numbers reproduced using IDB Analyzer applying student weight)

Although high civic knowledge is highly associated with self-reported future civic engagement in the ICCS studies, the young Nordic pupils score on or below the international average on the expected political participation scales in both ICCS studies (Schulz et al. 2018, p. 103). As visualized in Fig. 1.1, concerning civic attitudes, Nordic 14-year-olds endorse gender equality at significantly higher rates than the international average (Schulz et al. 2018, p. 126) and this is stable across the two time points. Endorsement of equal rights for all ethnic and racial groups are slightly lower than the international average in Denmark, on the international average in Finland, and slightly higher than the international average in Norway and Sweden (Schulz et al. 2018, p. 128).

### Social Background and Education Processes Associated with Strong Learning Outcomes

There are two main processes in which civic competences (knowledge, skills, attitudes, values, and dispositions) are said to be learned: (1) through participation, and (2) through knowledge acquisition (Hoskins and Janmaat 2019). The ICCS study focuses on measuring the participatory processes of learning and uses known measures of effective practice: open classroom climate and experiences of democracy in schools such as debates organized at school, school councils, and general involvement in decision making about how the school is run. The results from ICCS 2009 show strong associations between these learning methods and students’ intended political engagement in the form of voting, legal protest, and formal political activities (join a political party, trade union, volunteer for a party, or stand as a candidate) in Sweden (Hoskins and Janmaat 2019). Although the importance of an open classroom climate is documented, some of the classroom activities significantly increase the odds with which students achieve high civic knowledge more than others (Huang and Biseth 2016).

Socioeconomic status (SES) has frequently been associated with high levels of civic competence. In Sweden in 2009, participating in the above-mentioned learning activities was associated with socioeconomic background—the association was larger for countries like England and Ireland and smaller than Sweden for countries like Italy and Poland. The effect in Sweden, like England and Ireland, was at both the individual level and the school level. This means that the young people who go to schools with more disadvantaged young people in Sweden are reporting less open classroom climate experiences compared to schools with a higher level of advantaged student intake (Hoskins and Janmaat 2019). On top of this, there is an individual effect, where more disadvantaged young people within a school are reporting less of this experience compared to their more advantaged peers within the same school (Hoskins and Janmaat 2019).

In addition, undertaking education experiences can have different effects for different social groups and there is sometimes hope that an education experience can compensate for social disadvantage (Campbell 2008). From the participatory methods measured in the IEA citizenship datasets, none of these have been found so far to compensate for social disadvantage in Sweden, using the CIVED 1999 data (Persson 2015) or the ICCS 2009 data—although the subject citizenship education was found to be effective in compensating for disadvantage in England’s longitudinal data (Hoskins and Janmaat 2019). Chapter 10.1007/978-3-030-66788-7_5 of this book will investigate the effects of social background and participatory learning methods on the learning of civic competence for all four Nordic countries in ICCS 2009 and 2016.

### Country Specific Findings from Previous Analyses

Each of the four Nordic countries produced national reports that highlight specific findings from the ICCS 2016 data based on the countries’ particular interests, including trends when comparing with the ICCS 2009 data. The Danish national reports (Bruun et al. 2017, 2018) point out that the open classroom climate in Denmark is perceived by the pupils as very high but it has reduced since the ICCS 2009 study. Danish pupils additionally discuss political matters extensively at home. Danish students understand a good citizen as expected to obey the law, secure the family’s financial situation, and respect authorities. Interestingly, Danish young people do not consider engagement to protect human rights and the environment as important for adult citizens (Bruun et al. 2017, p. 3).

The Finnish national report shows that besides the general high scores in civic knowledge, students whose home language was the same as the ICCS test language had a higher score on civic knowledge compared to students with another home language. Additionally, the higher the SES, the higher the score on the knowledge component of the ICCS 2016 test (Mehtäläinen et al. 2017, p. 88). For the Finnish young people, traditional media such as newspapers are no longer the primary source of information, instead young people engage in discussions about political and social topics with both their parents and friends. Moreover, a sustainable environment seems to be one of the most engaging topics for students. Taken as a whole, there appears to be a slight increase from 2009 to 2016 in the level of participation and willingness to participate by Finnish youth, and the girls were slightly more active than the boys (Mehtäläinen et al. 2017, p. 89).

The Norwegian national reports show that compared to 2009, 14-year-olds in 2016 have higher civic knowledge achievement, higher institutional trust (Huang et al. 2017), more active civic engagement, increased positive attitudes toward the rights of ethnic minorities and immigrants (Hegna 2018a, b), higher intentions for electoral participation, and higher scores in considering a good citizen as one who obeys the law and respects authorities (Huang et al. 2017). There is a civic knowledge gap between 14-year-olds depending on the socioeconomic background such as parents’ educational attainment and native versus minority languages spoken at home, but the gap has been significantly reduced from ICCS 2009 to ICCS 2016 in Norway (Huang et al. 2017, pp. 54–72). Meanwhile, students with migrant background in Norway have become more active than the non-migrant students do in political and civic engagement in 2016 (Hegna 2018a). Further analyses of the Norwegian data show that students’ civic knowledge achievement is significantly correlated with their achievement in mathematics and language literacy (Seland and Huang 2018); and that student citizenship efficacy and current civic engagement have stronger association with student future intended political participation than civic knowledge does (Ødegård and Svagård 2018). They also report on a conducive democratic school environment with an open classroom climate and participation in the election of school councils and/or representatives (Huang et al. 2017).

The Swedish national report identifies an increase among teenagers in discussing political issues with both their parents and peers (Skolverket 2017). They also consider the classroom climate in school to be open to discussions and debates. Surprisingly, compared to much of the evidence in the field (Hoskins and Janmaat 2019; Keating and Janmmat 2016; Hoskins et al. 2011b), the report suggested the impact of democratic activities in school, such as the election of representatives for school councils, to be relatively low (Skolverket 2017).

## Complacency in Wealthy and Established Democracies?

The most surprising and consistent pattern found in the Nordic countries is the high levels of civic knowledge scores coupled with low current and expected future civic engagement and participation in comparison to the international average in the ICCS study. This is particularly puzzling since Nordic countries have consistently held some of the highest levels of democratic participation of the adult population in Europe (Hoskins and Mascherini 2009), and indeed the world (EIU 2020).

It could be possible to think that high scores on knowledge would equally lead to a high level of engagement, or that knowledge would have an impact on civic attitudes. The national coordinator of the ICCS 2016 study in Finland claims:

Finnish teenagers, like their Nordic peers in general, have excellent cognitive and attitudinal basic competences for participation, but most of these teenagers lack the interest and need for more active participation. They are happy with living in a steady representative democracy with functional safety networks. (Finnish Institute for Education Research 2017)

These patterns are similar to those found in ICCS 2009 (Hoskins et al. 2015) and CIVED 1999 (Hoskins et al. 2011a): Longer and more stable democracies combined with economic prosperity and in countries where teachers tended to prioritize critical thinking within citizenship education were found to develop higher levels of civic knowledge and skills and positive attitudes towards gender equality (Hoskins et al. 2015). In contrast, poorer and less stable democracies, in particular those where the teachers prioritized rights and responsibilities, were found to motivate young people to wish to politically engage (Hoskins et al. 2011a, 2015).

Based on ICCS 2016 and previous citizenship studies, there is a certain level of complacency among 14-year-olds in Nordic countries who do not find engagement in society particularly important or necessary to protect or ensure a sustainable democracy. The country-level data questions if there is an association at the individual level between civic knowledge and civic engagement in the Nordic countries. These associations remain at the individual level as Amnå and Zetterberg (2010) suggest after comparing the same cohort from CIVED with the European Social Survey data, Nordic youth are much more realistic in terms of future participation levels and similar numbers go on to participate whilst in other regions the actual numbers of young people who participate drops significantly. Ødegård and Svagård (2018) conclude, based on data from ICCS 2016 in Norway, that students’ level of democratic knowledge does not seem to influence their potential for future political engagement. Sætra and Stray (2019b), analyzing data from educators in ICCS 2016 in addition to 23 qualitative interviews conducted at the same time, question if students are provided with space in school to practice democratic engagement. They assert that teachers are more engaged in promoting independent and critical thinking than civic action and democratic engagement. Despite the opportunities available for students’ democratic participation in school, educators seem to focus their education on the knowledge component, and not nurture the possibilities for civic engagement in school (Biseth 2011).

Using Swedish data, Amnå and Ekman (2013) challenge the alleged passivity of youth by investigating different understandings of what is judged to be a passive citizen. They claim that a group of youth who are non-active are yet alert or on standby, ready to become active whenever they realize they can make an impact. In other words, the active/passive dichotomy, as discussed by Amnå and Ekman (2013), defies some of the suppositions implicit in the ICCS study and, more importantly, in terms of the realities of young people. However, there are many opportunities in Nordic countries for young people to participate in both schools and civil society during their later teens, which may provide the more crucial learning of political engagement practices for democratic societies. These opportunities also move beyond traditional ways of understanding engagement and include, for example, the use of digital and social media (see e.g., Sevincer et al. 2018). This is not yet effectively reflected when determining civic engagement in ICCS 2016. Complacency when it comes to civic engagement may seem, to some extent, present in the Nordic countries, but a study measuring new and alternative ways of engaging in democracy among youth, and the role of education in it, is not yet developed.

## The Positioning of This Book

Building on the context provided above, the following chapters explore essential themes about democracy, and civic and citizenship education in a particular Nordic context, being aware that the topics are by no means solely significant to a Nordic situation. Yet, a Nordic education model is claimed to exist with a free, comprehensive, and unified school system bringing together students from different socioeconomic strata with the aim of increased social mobility and democracy, and with a welfare state model at the centre (Imsen et al. 2017; Buchardt et al. 2013). The chapters of this book are based on themes typically engaging Nordic scholars or scholars engaging in studies of the Nordic countries with new analyses of the ICCS data. The main themes that the book addresses are: Nordic students’ understandings of citizenship, Nordic principles understandings of the priorities of citizenship education, digital citizenship education, environmental citizenship education, and inequalities and citizenship education.

Table 1.3 presents the numbers of cases and distributions of background variables used in the analyses presented in this volume. All Nordic data from ICCS 2009 and 2016 are of good quality fulfilling the IEA required technical standards, while authors of each chapter clarify their own analytical strategies and choices based on the appropriateness of specific methods in answering the research questions identified.

**Table 1.3:** An overview of numbers of cases and distributions of background variables from four Nordic countries using ICCS 2009 and ICCS 2016 data

	Denmark		Finland		Norway		Sweden		Total	
	2009	2016	2009	2016	2009*	2016	2009	2016	2009	2016
Number of schools	193	184	176	179	129	148	166	155	664	666
Number of school principal participants	171	181	174	174	118	142	155	141	609	630
Number of student participants	4408	6254	3307	3173	2926	6271	3464	3264	14105	18962
Average age of students	14.9	14.9	14.7	14.8	14.7	14.6	14.8	14.7	14.8	14.8
% of female students	52.6 (0.8)	51.3 (0.8)	51.1 (1.0)	47.4 (1.1)	50.0 (1.0)	49.5 (0.6)	50.1 (0.9)	49.3 (1.0)	51.0 (0.5)	49.4 (0.4)
% of students with migrant background (1st & 2nd generation) (standard error)	8.7 (0.8)	8.6 (0.8)	2.4 (0.5)	3.5 (0.5)	10.0 (1.0)	11.4 (1.1)	13.9 (1.2)	18.1 (1.6)	8.7 (0.5)	10.4 (0.5)
% of students having parents having a university degree (standard error)	22.6 (1.1)	24.8 (1.0)	32.6 (1.1)	41.8 (1.1)	50.2 (1.6)	59.6 (1.2)	52.0 (1.5)	58.9 (1.0)	39.3 (0.7)	46.3 (0.5)

*Notes* Percentages and means are calculated using the IDB Analyzer applying total weight of students. *Norway participated in ICCS 2009 with two student samples, i.e., grade 8 and grade 9, here we use the sample of grade 9 since Norway participated in ICCS 2016 with students of only grade 9

In the following sections are brief introductions of the analytical chapters and their specific positions within both the meaningful structure of this volume and the ongoing academic discourse.

### Contesting the Understanding of Civic Engagement

Bruun and Lieberkind, authors of the chapter *The Reserved Young Citizens of the Nordic Countries* (Chapter 10.1007/978-3-030-66788-7_2 in this volume), elaborate on the concept of “the standby citizen” by Amnå and Ekman (2013) when they develop the analytical category of “the reserved citizen.” They argue, based on data from ICCS 2009 and 2016 studies, that Nordic youth are not passive, but knowledgeable, have inclusive values, and are engaged in discussions with family and peers, yet they actively choose not to take part in more conventional political activities. In other words, Bruun and Lieberkind maintain that despite their reticence, the youth are pragmatic, reflective, critical thinkers who uphold democratic attitudes and values.

Seland, Huang, Arensmeier, Bruun, and Löfström, authors of the chapter *Aims of Citizenship Education Across Nordic Countries: Comparing School Principals’ Priorities in Citizenship Education 2009*–*2016* (Chapter 10.1007/978-3-030-66788-7_3 in this volume), investigate how principals in the four Nordic countries prioritize civic and citizenship education, using ICCS 2009 and 2016 data in their analysis. They find a strong and common Nordic priority on critical and independent thinking as democratic virtues among educators. As critical thinking seems to intertwine with civic knowledge, educators’ encouragement in students’ political engagement seems to be given less priority. This can be an essential matter in measuring youth’s political engagement or prediction of their future engagement: to what extent is this kind of engagement stimulated in school. ICCS 2009 and 2016 do contain data on educators, but these data are only analyzed to a limited extent with students’ results in the same study because the methodological design of the teacher survey only allows for analysis at school level. This chapter addresses this gap (Westheimer 2015; Sætra and Stray 2019b). One problem with the ICCS studies, at least in the Nordic countries, is the limited number of research reports from the dataset on educators’ responses (Eriksen and Huang 2019; Hu and Huang 2019; Sætra and Stray 2019a, b). The students’ results are expected to be, at least partially, an outcome of the schools’ efforts. This chapter addresses the question, what kind of citizens do the educators want to promote and how do they do this? Understanding students’ civic engagement is also about understanding teachers’ civic engagement in addition to their understanding of democracy per se and their capability to translate this into their teaching and learning activities (Biseth and Lyden 2018).

An additional element complicating the understanding of civic engagement further is the ever-growing presence of social media, particularly for young people. Being digitally literate is considered important for a democratic citizen (see e.g., Fraillon et al. 2014; Frau-Meigs et al. 2017). Yet, the civic realities of today are not necessarily aligned with civic habits of the past (Papacharissi 2010). Traditional media such as newspaper and TV no longer have the same importance in young people’s lives (Schulz et al. 2018). Young people keep informed through the internet and social media. Moreover, social media provides a low threshold for political participation and civic engagement. Young educators well versed in the use of social media, however, tend to struggle with using social media for civic and citizenship education purposes (Biseth et al. 2018; Gudmundsdottir and Hatlevik 2018).

In Chapter 10.1007/978-3-030-66788-7_4 in this volume, *Developing Digital Citizenship and Civic Engagement Through Social Media Use in Nordic Schools*, Christensen, Biseth, and Huang discuss results from ICCS 2016 compared with the four participating Nordic countries’ core curricula. When data collection for the ICCS 2016 study took place, core curricula were in place promoting active digital citizenship. However, the curricula were not sophisticated in this regard.^1^ The Nordic educators reported technically well-equipped schools and staff able to use digital tools, creating a potential for developing digital citizenship, yet teachers and students reported their rather limited use of social media for civic and political engagement both in and outside of school. Despite the youth using social media extensively for entertainment, it nevertheless appears less interesting to use social media in school as a place for civic engagement and democratic activities, making school detached from the world of the youth.

### Democratic Dispositions in Nordic Democracies

As democracy is characterized by a set of values by which we organize our society, how such values manifest themselves in everyday praxis is of importance (White 1996). Biesta (2006) argues that students cannot become democratic if schools do not practice democratic ideals and that these ideals need to permeate all activities in school (Biesta 2006). The democratic dispositions present in staff and students in school may tell us about the democratic qualities of a school. As the school is educating future citizens, the presence or lack of democratic dispositions may provide indications of the future of the Nordic democracies or at least areas that need our attention. The chapters in this book make use of data from the ICCS studies in 2009 and 2016 to analyze and discuss different aspects important to Nordic countries—and beyond.

In Chapter 10.1007/978-3-030-66788-7_5 of this volume, *Socioeconomic Inequalities in Civic Learning in Nordic Schools: Identifying the Potential of In*-*school Civic Participation for Disadvantaged Students,* Hoskins, Huang, and Arensmeier raise what is to some extent an uncomfortable topic on what we believe to be our Nordic democratic dispositions. The authors investigate if there are social inequalities in the levels of skills needed to politically engage in Nordic countries and identifying the role of school in either reducing or increasing inequalities in civic competence. Socioeconomic inequalities are found, most visibly in Sweden, but significant in all Nordic countries, and stable across ICCS 2009 and 2016. Some learning experiences are not equally accessible to all socioeconomic groups in school, making the school a contributor in upholding socioeconomic inequalities. In other words, developing civic competences and educating citizens for a democratic society currently varies based on your socioeconomic background. Schools and educators need to ensure developing democratic dispositions and incarnate democratic values, also in a pluralistic society (White 1996; Biesta 2006).

Following a similar thread, Huang and Cheah present a picture of Nordic student environmental citizenship divided by SES and gender in Chapter 10.1007/978-3-030-66788-7_6, titled *The Young Environmental Citizens in Nordic Countries: Their Concerns, Values, Engagement, and Intended Future Actions.* The authors investigate if the Nordic “Greta Thunberg generation” of 14-year-olds in 2016 have similar or different concerns, values, engagement, and intended actions regarding environmental issues in comparison with their European and international peers and if there are socioeconomic inequalities of student environmental citizenship in the Nordic countries as well. Among the indicators of environmental citizenship from ICCS 2016 data, Nordic students stand out with their high concerns of pollution and climate change as the two biggest threats to the future of the world, in comparison with their European and international peers. While there are significant differences between countries, Nordic students as a whole are somehow lower in indicators of values, engagement, and intended actions of environmental citizenship, than the European and international averages. The analyses find that significant inequalities of student environmental citizenship exist between high and low SES and between boys and girls and there is a significant interaction effect between socioeconomic background and gender in all Nordic countries. However, socioeconomic background and gender have much less effect among students with migrant background than among students without migrant background. Eventually, student environmental citizenship is socially divided by socioeconomic background and by gender.

## Conclusion

The themes presented in this book are not exhaustive of the interest for civic and citizenship education in the Nordic countries. However, this current volume represents the first time ICCS data from the Nordic countries are analyzed and compared across themes and countries. Moreover, the chapters are proposed and authored by Nordic scholars, with a valued British colleague on the team. We hope this Nordic endeavor and lenses will supplement previous analyses that have focused mostly on individual national results of ICCS in this region. Today, only a limited number of academic works are published based on ICCS beyond the national reports, e.g., comparing national data of ICCS 2009 and 2016 (Hegna 2018a, b; Stray and Huang 2018) or comparing ICCS 2016 results across countries on student attitudes (Huang et al. 2018) and on school and teacher variables (Cheah and Huang 2019; Eriksen and Huang 2019; Hu and Huang 2019). The topics included in this book are relevant for policymakers, researchers, school principals, and teachers who are working and interested in the Nordic models of civic and citizenship education and democracy. These topics have become ever more important now when all countries in the globe are facing the aftermath of the COVID-19 pandemic (Worldometers 2020) which has its impact on almost every aspect of our society. Three topics presented in this book have been central in the Nordic educational systems, which we hope to provoke thinking of amongst the readers. First, although sustainable development has received a central position in the Nordic core curricula, the topic of youth political participation and civic engagement and how it can best be fostered and facilitated by our school education has been less so, yet it is essential for a functional democracy and a sustainable future. Second, although Nordic education systems are based on the principles of equality, there are persistent effects of social inequality on student educational achievement and how schools play a role in enhancing and mitigating this effect needs to continue to be on countries’ policy agenda. Third, the Nordic reality is that our current school students are reserved, digital, and environmental citizens and the implications for this for the future of the Nordic democratic institutions is an ongoing question. This makes it ever more crucial to ensure educational systems that can cope, support, and develop all young Nordics to create a sustainable democratic world.

## Footnotes

1 It must be noted that curricular changes have taken place in all countries after ICCS 2016, increasing the focus on ICT skills in general and in relation to a democratic citizen.
